# Chromosome 3q29 deletion with gastrointestinal malformation: a case report

**DOI:** 10.1186/1752-1947-5-285

**Published:** 2011-07-05

**Authors:** Ma'in Masarweh

**Affiliations:** 1 King Hussein Cancer Centre, Amman, Jordan

## Abstract

**Introduction:**

Most chromosome 3 deletions are associated with neuro-developmental and eye abnormalities. Here, we report a rare and unusual multiple congenital abnormality, including ano-rectal malformation, in conjunction with chromosome 3q29 segment deletion, which has not previously been reported.

**Case presentation:**

A three-month-old female Jordanian baby presented with an absent anus and corneal opacities and was referred for further management after a diverting colostomy operation at the age of one day.

**Conclusion:**

Chromosome 3q29 deletion is associated with additional abnormalities to neurological ones, such as ano-rectal malformations. We need to investigate a patient fully to find such hidden clinical features.

## Introduction

Chromosome 3q deletion syndrome, 3q-syndrome, and monosomy 3q are all synonyms of the same clinical description, and it is considered an uncommon anomaly [1]. This condition is not associated with any antenatal abnormalities, and the birth history is uneventful in most patients [2]. Deletion of the long arm of chromosome 3 may present with variable phenotypes, consisting mainly of microcephaly, unusual facial appearance, eye abnormalities, deformed ears, and a delay in growth and development. A partial deletion of the long arm of chromosome 3, mainly the q23-q25 and q22-q23 bands, is associated with blepharophimosis-ptosis-epicanthus inversus syndrome (BPES), and most reported cases are linked to this deletion [3].

The major phenotypic features involve nearly all of the systems, including the head and neck (microcephaly, dolichocephaly, trigonocephaly, retro-micrognathia, large abnormally shaped posteriorly rotated and low-set ears, prominent or beaked nose, broad nasal bridge, and cleft lip and palate), the spine (13 thoracic vertebrae and scoliosis or kyphosis), the heart, and the nervous system [4]. It has been reported that some deletions are hereditary, such as the 3q23-q25 deletion, and some of them may be associated with female infertility [3,5].

## Case presentation

**Figure 1:**
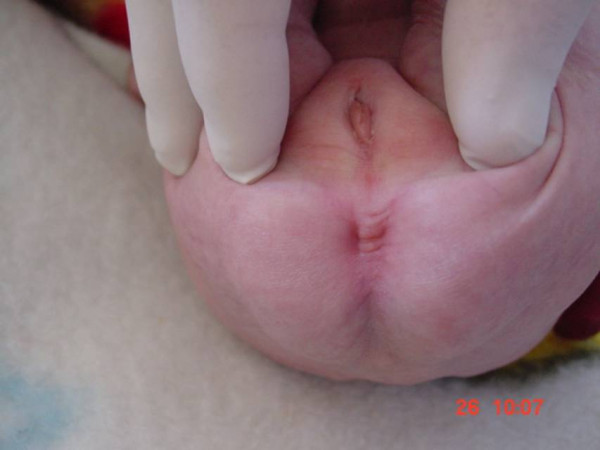
**Absent anus**.

A three-month-old Jordanian female baby was vaginally delivered at full-term to a 25-year-old mother on her second pregnancy, the first being successfully carried past 20 weeks (G2P1). The baby's parents were healthy and non-related. She presented with a history of ano-rectal malformation for further management. She underwent sigmoid loop colostomy after 24 hours of birth outside our hospital. General examination of the baby upon presentation showed a few external abnormal features such as a small head, low-set ears, corneal opacities in both eyes, and a high-arched palate in addition to signs of dehydration and being underweight. An abdominal examination revealed ulcerated inflamed skin in the left lower quadrant around a prolapsing loop colostomy, with fluid stool content. A perineal examination showed a single orifice, with urine coming through it. No anal or vaginal orifices were seen (Figure 1), and the labio-scrotal folds were under-developed. Her work-up showed that she was suffering from atrial septal defect (ASD), ventricular septal defect (VSD), a delay in mental development, corneal opacities, and cloacal ano-rectal malformation.

**Figure 2:**
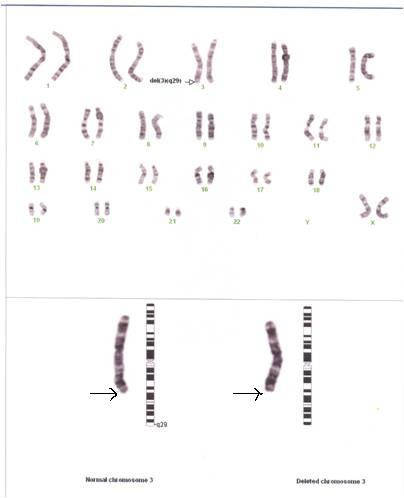
**Chromosomal analysis**.

A micturating cysto-urethrogram confirmed the common channel of the deformity. Ultrasonography of the abdomen was normal. Skeletal survey and brain magnetic resonance imaging results were normal. A chromosomal analysis was carried out, involving 20 CTG-banded cells from two cultures, and five cells were karotyped and photographed, showing 46,XX,del(3)(q29) (Figure 2).

Our patient was treated for the colostomy complication and, at the age of nine months, she underwent full repair of the cloaca through posterior saggital ano-recto-vaginoplasty. Smooth post-operative recovery was achieved, and three months later the colostomy opening was closed. Her heart condition was stable over the three years of follow-up, the VSD and ASD reduced in size, her corneal opacities did not progress, and she remained under the supervision of our ophthalmologist. Her mental development continued to grow slowly, and she is now able to speak a few words and stand up without support.

## Discussion

Chromosome 3 deletions are rare anomalies [6], with deletions involving band 2 being more commonly reported, as mentioned by most reviewed reports. We found some case reports mentioning the association of this abnormality with the development of congenital diaphragmatic hernia [6,7] and enlarged penile size [8], in addition to the recognizable contiguous gene syndrome of deletion of 3q23 in BPES [9]. Another report by Cai *et al*. [10] described the unusual unilateral ptosis and absence of the epicanthus inversus, but the chromosomal study showed unbalanced translocation, 46,XX,der(7)t(3;7)(q26-qter;q+), which resulted in trisomy for distal 3q.

Another report described new features of the deletion of 3q with progressive scoliosis, multiple skin pigmentations, and renal abnormalities [11]. A deletion similar to that in our patient was found in a case reported by Baynam *et al*. [12] but with different clinical features.

Here, our patient had an ano-rectal anomaly in addition to corneal opacities, which we could not find in any of the reports reviewed, including Ballif *et al*. in 2008 [4] or Willatt L *et al*. in 2005, which described the most up-to-date cases of 3q29 microdeletions in six patients with different phenotypic features [2]. It seems that these deletions are not consistent with any one type of clinical abnormality, and there might still be some other molecular factors that play a role in the development of the clinical symptoms of chromosome 3 deletions. Additional investigations using high resolution techniques, such as single nucleotide polymorphism or oligo arrays, are advised to confirm pure deletion of 3q29 and to exclude, for example, a chromosome translocation, and thus duplication of another chromosome adding to the phenotype. Unfortunately, these techniques are not available at our institution or in our country.

Our patient had been followed up for three years. Her development was very slow as compared to her sister, who was born two years later and who did not show any similar abnormality.

## Conclusion

Chromosome 3q29 deletions are associated with other abnormalities such as ano-rectal malformations, and not only the previously reported neurological abnormalities. We need to investigate a patient fully to find such hidden clinical features. The next step is to understand the exact molecular mechanisms of this disease. More research is required to identify if there is a mode of inheritance in chromosome 3 deletions, as in BPES. This will help to explain why these patients vary in their clinical features.

## Consent

Written informed consent was obtained from the patient's father for publication of this case report and any accompanying images. A copy of the written consent is available for review by the Editor-in-Chief of this journal.

## Competing interests

The author declares that they have no competing interests.
